# Limiting the caesarean section rate in low risk pregnancies is key to lowering the trend of increased abdominal deliveries: an observational study

**DOI:** 10.1186/1471-2393-12-3

**Published:** 2012-01-09

**Authors:** Ilse Delbaere, Hendrik Cammu, Evelyne Martens, Inge Tency, Guy Martens, Marleen Temmerman

**Affiliations:** 1 Ilse Delbaere: Department of Obstetrics and Gynaecology, Ghent University Hospital, Belgium; 2 Department of Obstetrics and Gynaecology, Free University Brussels, Brussels, Belgium; 3 Study Centre for Perinatal Epidemiology, Brussels, Belgium; 4 Department of Obstetrics and Gynaecology, Ghent University Hospital, Belgium; 5 Study Centre for Perinatal Epidemiology, Brussels, Belgium; 6 Department of Obstetrics and Gynaecology, Ghent University, Belgium

## Abstract

**Background:**

As the rate of Caesarean sections (CS) continues to rise in Western countries, it is important to analyze the reasons for this trend and to unravel the underlying motives to perform CS. This research aims to assess the incidence and trend of CS in a population-based birth register in order to identify patient groups with an increasing risk for CS.

**Methods:**

Data from the Flemish birth register 'Study Centre for Perinatal Epidemiology' (SPE) were used for this historic control comparison. Caesarean sections (CS) from the year 2000 (N = 10540) were compared with those from the year 2008 (N = 14016). By means of the Robson classification, births by Caesarean section were ordered in 10 groups according to mother - and delivery characteristics.

**Results:**

Over a period of eight years, the CS rise is most prominent in women with previous sections and in nulliparous women with a term cephalic in spontaneous labor. The proportion of inductions of labor decreases in favor of elective CS, while the ongoing inductions of labor more often end in non-elective CS.

**Conclusions:**

In order to turn back the current CS trend, we should focus on low-risk primiparae. Avoiding unnecessary abdominal deliveries in this group will also have a long-term effect, in that the number of repeat CS will be reduced in the future. For the purpose of self-evaluation, peer discussion on the necessity of CS, as well as accurate registration of the main indication for CS are recommended.

## Background

The Caesarean section (CS) rate has been rising through the twentieth century with a substantial increase in the last 30 years. Currently, a Caesarean section rate of 18-20% is recorded in developed countries and is more than 30% in the United States alone [1,2]. These rates far exceed the 15% that is recommended by the World Health Organization [3]. It is therefore of interest to bring attention to this rising CS-rate, and to better understand the increased pathology in pregnancy and delivery of women in which a uterine scar has been registered. Examples of such pathology are a higher risk of spontaneous abortion, abruption, placenta previa, placenta accrete and rupture [3,4]. Although the risk for maternal death is low in developed countries and maternal mortality associated with CS has decreased substantially over time, the hazard of this obstetrical outcome is still doubled (or more) after (elective) CS [4,5]. In addition, abdominal delivery goes hand in hand with a higher risk of operative complications (infections, haemorraghia, visceral injury, thromboembolism) and psychological problems in the postpartum period [3,4].

Our research aims to assess the incidence and trend of CS in a low-risk population and to better understand the rationale of the increase of abdominal deliveries in this population. Only when these questions are answered, can there be approaches instituted to level or adjust the rising trend in CS rates.

## Methods

The Study Centre for Perinatal Epidemiology (SPE) is a birth register which collects data on every birth in Flanders (Northern and Flemish speaking region of Belgium) [6]. This data collection occurs anonymously and is population-based, as all 72 Flemish maternity units cooperate. Next to permanent registration of perinatal data, another important objective of the SPE is evaluation of perinatal care in order to inform hospitals about their policy and to stimulate quality of care improvement. Registration started in 1987, in December 2009 there were in total 1,378,873 deliveries of children with a birth weight of minimum 500 grams included in the database. For this study, we included all live born children (birth weight > 500 grams) delivered by caesarean section in the year 2000 or 2008. Data from 2004 are included in order to monitor for deviations from the trend within this timespan.

The study design has been approved from the Ethical Committee of the University Hospital Ghent.

In order to range the SPE-population in subgroups, the Robson classification was used (Robson 1996). Within the Robson 10-group classification, Caesarean sections are ordered according to characteristics of patients and their deliveries, rather than to the indication for CS (see table 1). As such, the different obstetric populations requiring CS are classified and comparison of CS rates among these populations is possible. This classification is engaging, in that all possible deliveries are prospectively identifiable in order to improve outcomes in the same patients in the future; furthermore the classification is totally inclusive and mutually exclusive. Obstetric concepts and parameters used to group women in the ten-group classification are: category of pregnancy, previous obstetric record, course of labor and delivery and gestational age [7].

**Table 1:** The Robson 10 - group classification

Group 1	Nulliparous, single cephalic, ≥ 37 weeks, in spontaneous labor
Group 2	Nulliparous, single cephalic, ≥ 37 weeks, induced or CS before labor
Group 3	Multiparous (excluding prev. CS), single cephalic, ≥ 37 weeks, in spontaneous labor
Group 4	Multiparous (excluding prev. CS), single cephalic, ≥ 37 weeks, induced or CS before labor
Group 5	Previous CS, single cephalic, ≥ 37 weeks
Group 6	All nulliparous breeches
Group 7	All multiparous breeches
Group 8	All multiple pregnancies (including prev. CS)
Group 9	All abnormal lies (including prev. CS)
Group 10	All single cephalic, ≤ 36 weeks (including prev. CS)

With the use of Robson classification, only SPE - data from 2000 on could be included, since 'history of a previous CS' was included as a variable in the database from 2000 onwards.

Elective Caesarean section is defined as a section which was planned before admission of the patient to the hospital (pre-labor section). A non - elective Caesarean section is a section for which there were initially no indications, but CS occurred due to complications after onset of labor (CS in labor).

## Results

### Patient characteristics

Relevant and available characteristics of patients, their pregnancy and delivery are depicted in table 2. Although the percentage of multiple pregnancies is comparable in both study years, the incidence of malpresentations is higher in 2008 (5.9% versus 4.9% in 2000). Mean gestational age at birth remained stable at 38.7 weeks in both years. Whereas no differences in mean birth weight are found throughout the years (3294 grams in 2000, 3309 grams in 2008), the percentage of vaginal deliveries (including vaginal breech deliveries) as well as vacuum extractions and the use of the forceps have all decreased in the study period in favor of Caesarean sections, with equal augmentations in primary CS and secondary CS. Even though the permillages of both fetal death and neonatal death are lower in 2008, no significant difference was found between both years. A lower incidence of multiple pregnancies is noticeable in 2004. This is due to the introduction of a government measure in 2003 in order to stimulate the use of single embryo transfer in assisted reproduction [8].

**Table 2:** Patient, pregnancy and delivery characteristics

	2000	2004	2008	OR (95% CI) 2000 - 2008
**Patient and pregnancy characteristics**	60993 women	61647 women	68199 women	

Primiparae aged 35 or older	5.2%	6.6%	7.6%	1.50 (1.43 - 1.57)***

Multiparae aged 35 or older	15.9%	18.4%	20%	1.32 (1.28 - 1.36)***

Parity				

First child	46.7%	47.8%	46.9%	1.01 (0.99 - 1.03)

Second child	34.2%	33.5%	34.9%	1.03 (1.01 - 1.06)**

Third child	12.7%	12.5%	12.2%	0.96 (0.92 - 0.99)**

Assisted Reproduction				

Assisted induction of ovulation	1.7%	2.1%	2.1%	1.24 (1.14 - 1.35)***

Assisted Reproductive Technology (*In vitro fertilisation and Intra Cytoplasmic Sperm Injection)*	1.7%	2.3%	3.3%	1.97 (1.83 - 2.13)***

Twin pregnancies	1.80%	1.61%	1.83%	1.02 (0.94 - 1.10)

Hypertension during pregnancy	4.9% (2002)	4.8%	4.8%	0.98 (0.93 - 1.03)

Diabetes during pregnancy	1.2% (2002)	1.4%	1.8%	1.51 (1.38 - 1.66)***

Induction of labor	30.3%	27.6%	25.3%	0.78 (0.76 - 0.80)***

Preterm birth (GA < 37 weeks)	7.1%	7.5%	7.4%	1.05 (1.00 - 1.09)*

**Delivery characteristics**	62 128 births	62657 births	69470 births	

Presentation				

Vertex	95.0%	94.3%	94.1%	0.84 (0.80 - 0.88)***

Breech	4.6%	5.2%	5.4%	1.18 (1.13 - 1.25)***

Other	0.3%	0.5%	0.5%	1.67 (1.39 - 2.01)***

Low birth weight (< 2500 gr)	6.8%	6.9%	6.9%	1.02 (0.97 - 1.06)

Vaginal cephalic delivery	71.2%	69.8%	69.3%	0.91 (0.89 - 0.94)***

Vaginal breech delivery	0.5%	0.5%	0.3%	0.60 (0.50 - 0.72)***

Vacuum extraction	11.2%	9.7%	9.5%	0.83 (0.80 - 0.86)***

Forceps	1.3%	0.9%	0.8%	0.61 (0.55 - 0.68)***

Caesarean sections	16.4%	18.3%	19.5%	1.23 (1.20 - 1.27)***

Elective C - section	9.2%	10.8%	11.2%	1.24 (1.20 - 1.29)***

Unplanned C - section	6.6%	7.4%	8.2%	1.26 (1.21 - 1.32)***

Perinatal mortality	6.7‰	6.4%	6.2‰	0.92 (0.80 - 1.06)

Foetal death	4.4 ‰	4.3‰	4.3 ‰	0.98 (0.83 - 1.16)

Neonatal death	2.3 ‰	1.8‰	1.9 ‰	0.83 (0.65 - 1.06)

*P < 0.05, **P < 0.01, ***P < 0

### The Robson 10 - group classification

Tables 3 en 4 shows the 10-group classification according to Robson. We compared the 10-groups for the years 2000 with those of the year 2008 in table 3. In order to picture the trend over those years, the classification of the year 2004 is provided in table 4. Columns 3 and 7 show the number of CS for the total number of births in each group for the years 2000 and 2008 respectively. The relative value of groups in total births (columns 4 and 8) is calculated by dividing the number of births in each group by the total obstetric population for the given year and expresses the 'importance' or 'weight' of each group in the total population. The next (fifth and ninth) column shows the CS rate in each group and represents the fractions as depicted in columns 3 and 7. The 'contribution per group' columns (columns 6 and 10) show the percentage contribution by each group to the overall birth rate (number of CS/total obstetric population for the given year). This classification allows assessing which groups contribute more to the overall section rate.

**Table 3:** Robson's 10-group classification of Flemish women giving birth in 2000 or 2008

		2000				2008				Δ Caesarean sections (%) (2008 - 2000)	Δ contribution per group (2008 - 2000)
**1**.	**2**.	**3**.	**4**.	**5**.	**6**.	**7**.	**8**.	**9**.	**10**.	**11**.	**12**.

**Columns/groups**		**Number of CS/Total births**	**Relative value group in total births (%)**	**Caesarean sections (%)**	**Contribution per group (%)**	**Number of CS/Total births**	**Relative value group in total births (%)**	**Caesarean sections (%)**	**Contribution per group (%)**		

**1**.	**Nulliparous, single cephalic, ≥ 37 weeks, in spontaneous labor**	1036/15316	24.7	6.8	1.7	1635/18571	26.7	8.8	2.4	2.0	0.7

**2**.	**Nulliparous, single cephalic, ≥ 37 weeks, induced or CS before labor**	2087/8843	14.2	23.6	3.4	2576/8793	12.6	29.3	3.7	5.7	0.3

**3**.	**Multiparous (excluding prev. CS), single cephalic, ≥ 37 weeks, in spontaneous labor**	233/16568	26.7	1.4	0.4	295/18688	26.9	1.6	0.4	0.2	/

**4**.	**Multiparous (excluding prev. CS), single cephalic, ≥ 37 weeks, induced or CS before labor**	804/9505	15.3	8.5	1.3	707/8451	12.2	8.4	1.0	-0.1	- 0.3

**5**.	**Previous CS, single cephalic, ≥ 37 weeks**	2127/3409	5.5	62.4	3.4	3372/5369	7.7	62.8	4.8	0.4	1.4

**6**.	**All nulliparous breeches**	1451/1642	2.6	88.4	2.3	1792/1882	2.7	95.2	2.6	6.8	0.3

**7**.	**All multiparous breeches**	765/1125	1.8	68.0	1.2	1049/1241	1.8	84.5	1.5	16.5	0.3

**8**.	**All multiple pregnancies (including prev. CS)**	1079/2253	3.6	47.9	1.7	1441/2558	3.7	56.3	2.1	8.4	0.4

**9**.	**All abnormal lies (including prev. CS)**	188/200	0.3	94.0	0.3	171/180	0.3	95.0	0.2	1.0	- 0.1

**10**.	**All single cephalic, ≤ 36 weeks (including prev. CS)**	770/3264	5.3	23.6	1.2	978/3800	5.5	25.7	1.4	2.1	1.4

	**TOTALS**	**10540/62125**			**17.0**	**14016/69533**			**20.2**		**3.2**

**Table 4:** Robson's 10-group classification of Flemish women giving birth in 2004

		2004			
**1**.	**2**.	**3**.	**4**.	**5**.	**6**.

**Columns/groups**		**Number of CS/Total births**	**Relative value group in total births (%)**	**Caesarean sections (%)**	**Contribution per group (%)**

**1**.	**Nulliparous, single cephalic, ≥ 37 weeks, in spontaneous labor**	1214/16806	26.8	7.2	1.9

**2**.	**Nulliparous, single cephalic, ≥ 37 weeks, induced or CS before labor**	2298/8509	13.6	27.0	3.7

**3**.	**Multiparous (excluding prev. CS), single cephalic, ≥ 37 weeks, in spontaneous labor**	238/16041	25.6	1.5	0.4

**4**.	**Multiparous (excluding prev. CS), single cephalic, ≥ 37 weeks, induced or CS before labor**	718/8521	13.6	8.4	1.1

**5**.	**Previous CS, single cephalic, ≥ 37 weeks**	2739/4310	6.9	63.5	4.4

**6**.	**All nulliparous breeches**	1560/1650	2.6	94.5	2.5

**7**.	**All multiparous breeches**	823/1115	1.8	82.8	1.5

**8**.	**All multiple pregnancies (including prev. CS)**	1075/2029	3.2	53.0	1.7

**9**.	**All abnormal lies (including prev. CS)**	168/178	0.3	94.4	0.3

**10**.	**All single cephalic, ≤ 36 weeks (including prev. CS)**	889/3501	5.6	25.4	1.4

	**TOTALS**	**11818/62654 (18.9%)**			

Our results show an increase of the CS rate in all groups, except in the group of multiparous women with a term single cephalic pregnancy (group 3) which remains more or less stable. The most notable rise is detected in women with previous sections (group 5) and in nulliparous term women in spontaneous labor of a single cephalic (group 1). In multiple pregnancies (group 8), there is an obvious lower threshold in 2008 to perform a CS compared with the year 2000. In 2000, groups 2, 5 and 6 contribute most to the CS- rate; in 2008 the dominance of group 5 (repeat CS) is notable, the group of nulliparous women with induction or elective CS is the second - most important and the third place is shared by group 1 (lowest - risk group) and group 6. As a consequence the group of term nulliparous women with a singleton cephalic in spontaneous labor contribute as much to the CS - rate as the group with nulliparous breech presentations. Conversely, there is no 100% CS - rate in group 9 (abnormal lies), this can be explained by the high preterm birth rate in this group. Groups 1 to 4 represent uncomplicated pregnancies; in 2000 this group represented 80.9% of total deliveries, while in 2008 there was a slight increase (88.4%). This implicates a increase in the percentage of low - risk patients. When the magnitude of rise in contributions per group is calculated, a 41% rise is detectable in both groups 1 and 5.

**Figure 1:**
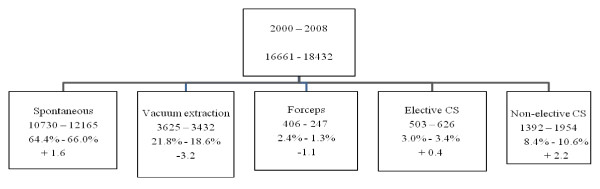
**Mode of delivery in low risk deliveries in Flanders (2000 and 2008)**.

**Figure 2:**
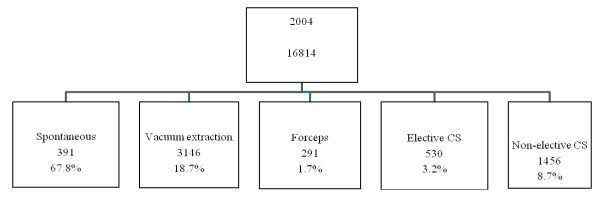
**Mode of delivery in low risk deliveries in Flanders (2004)**.

Details of mode of delivery in the low-risk populations are depicted in Figure 1 and 2 (for 2004). Low-risk deliveries are defined as deliveries in primipare women, who deliver from a term live born singleton without malformations in vertex presentation with a birth weight between 3000 and 4000 grams. Figure 1 shows an important reduction of instrumental deliveries in the advantage of natural vaginal deliveries, but more notably in favor of the non - elective CS - rate.

In table 5 CS - rates according to different maternal age groups are illustrated. There is a relative increase of the CS - rate in all age groups, but no statistical significant rise in risk - groups (teenage mothers or women of advanced age). In the age group 20-34 years, a significant increase of the CS - rate is found.

**Table 5:** Trend in maternal age (2000, 2004 and 2008, low risk patients only

	2000			2004			2008			OR (95% CI)
**Maternal age**	**Total deliveries**	**C - section**	**%**	**Total deliveries**	**C - section**	**%**	**Total deliveries**	**C - section**	**%**	

< 20 years	727	52	7.2	633	56	8.8	693	57	8.2	1.16 (0.77 - 1.75)

20-24 years	3829	377	9.8	3877	412	10.6	3776	476	12.6	1.36 (1.18 - 1.58)

25-29 years	8005	863	10.8	7846	853	10.9	8512	1072	12.6	1.19 (1.08 - 1.31)

30-34 years	3343	458	13.7	4116	619	15.0	4221	677	16.0	1.20 (1.05 - 1.37)

35-39 years	689	127	18.4	965	181	18.8	1073	241	19.6	1.08 (0.84 - 1.39)

≥ 40 years	68	18	26.5	132	38	28.8	157	57	36.3	1.58 (0.81 - 3.12)

The trend of inductions of delivery is depicted in table 6. In 2000, 33% of all deliveries were induced; in 2008 this proportion decreased to 27.7%. However, the percentage of CS after induction increased from 13.2 to 17.9%. Particularly in gestational weeks 39, 40 and 41, there was an increase in failure of induction.

**Table 6:** Trend in induction of delivery (2000, 2004 and 2008, low risk patients only)

	2000				2004				2008				OR (95% CI) 2004-2008
**Gestational age**	**Total deliveries**	**Induction**	**CS in induced deliveries**	**%**	**Total deliveries**	**Induction**	**CS in induced deliveries**	**%**	**Total deliveries**	**Induction**	**CS in induced deliveries**	**%**	

37 weeks	833	230	22	9,6	796	154	32	20.8	869	192	28	14,6	0.62 (0.33-1.17)

38 weeks	2486	659	79	12,0	2583	634	101	15.9	2903	657	103	15,7	0.73 (0.53-1.02)

39 weeks	4821	1244	135	10,8	5030	1070	144	13.5	5737	1062	163	15,3	0.67 (0.52-0.86)***

40 weeks	6823	2337	307	13,1	6702	1983	317	16.0	7058	1890	340	18,0	0.69 (0.58-0.82)***

41 weeks	2077	1077	177	16,4	2334	1273	215	16.9	2739	1499	307	20,4	0.76 (0.62-0.94)*

42 weeks	105	65	19	29,3	123	82	22	26.8	110	74	21	28,4	1.04 (0.47-2.32)

**Totals**	**17145**	**5612**	**739**	**13,2**	**17568**	**5196**	**831**	**16,0**	**19416**	**5374**	**962**	**17,9**	

### Comparison between the hospital with lowest Caesarean section rate and the hospital with highest Caesarean section rate

Differences in CS rates between the hospital with the lowest rate of abdominal delivery (hospital A) and the hospital with the highest rate (hospital B) are depicted in table 7. In general, there is a three times higher Caesarean section rate in the hospital with the highest rate (which is not a university hospital) when compared to the hospital with the lowest rate (26.3% versus 9.1%); looking at the low-risk pregnancies only (groups 1 and 2), a five times higher CS rate is found (24.6% versus 4.6%). Because of the historically low CS rate in hospital A, the relative value of group 5 is considerably lower in this hospital when compared with hospital B (1.6% versus 9.1%). The differences in CS percentage in the breech - presentation groups (6 and 7) are notable as well.

**Table 7:** Robson's 10 - group classification of hospital with lowest c - section rate (A) and hospital with highest c - section rate (B) in Flanders (2000 and 2008)

		Hospital A: lowest c - section rate (2000 - 2008)				Hospital B: highest c - section rate (2000 - 2008)				Δ Caesarean sections (%) (hospital highest rate - hospital lowest rate)	Δ contribution per group (hospital highest rate - hospital lowest rate)
1.	2.	**3**.	**4**.	**5**.	**6**.	**7**.	**8**.	**9**.	**10**.	**11**.	**12**.

**Columns/groups**		**Number of CS/Total births**	**Relative value group in total births (%)**	**Caesarean sections (%)**	**Contribution per group (%)**	**Number of CS/Total births**	**Relative value group in total births (%)**	**Caesarean sections (%)**	**Contribution per group (%)**		

**1**.	**Nulliparae, singleton, head presentation, ≥ 37 weeks, spontaneous labor**	77/2033	37.2	3.8	1.4	234/1575	23.1	14.9	3.4	11.1	2.0

**2**.	**Nulliparae, singleton, head presentation, ≥ 37 weeks, induction or primary section**	70/546	10.0	12.8	1.3	417/1228	18.0	39.7	7.1	26.9	5.8

**3**.	**Multiparae, singleton, head presentation, ≥ 37 weeks, spontaneous labor, except previous CS**	16/1614	29.6	1.0	0.3	22/1363	20.0	1.6	0.3	0.6	/

**4**.	**Multiparae, singleton, head presentation, ≥ 37 weeks, induction or primary CS**	48/564	10.3	8.5	0.9	89/1202	17.6	7.4	1.3	-1.1	0.4

**5**.	**Previous CS, singleton, head presentation, ≥ 37 weeks**	55/85	1.6	64.7	1.0	466/634	9.3	73.5	6.8	8.8	5.8

**6**.	**All breech presentations, nulliparae**	93/125	2.3	74.4	1.7	169/174	2.5	97.1	2.5	22.7	0.8

**7**.	**All breech presentations, multiparae**	43/91	1.7	47.3	0.8	108/129	1.9	83.7	1.6	36.4	0.8

**8**.	**All twins or HOM, previous CS inclusively**	53/158	2.9	33.5	1.0	116/191	2.8	60.7	1.7	27.2	0.7

**9**.	**All transverse presentations, previous CS inclusively**	7/8	0.1	87.5	0.1	17/18	0.3	94.4	0.2	6.9	0.1

**10**.	**All singletons, head presentations, ≤ 36 weeks, previous CS inclusively**	35/235	4.3	14.9	0.6	84/310	4.5	27.1	1.2	12.2	0.6

	**TOTALS**	**497/5459**			**9.1**	**1792/6824**			**26.3**		**17.2**

While there is a balanced contribution of the first 5 groups (4.9%) and the last 5 groups (4.2%) in hospital A, the balance in hospital B gravitates towards the lowest - risk groups (18.9% versus 7.2%). In hospital A, there was a perinatal death rate of 3.63 ‰, compared to 4.97 ‰ in hospital B.

### Trends in perinatal death

Fetal well-being is one of the main reasons to proceed to Caesarean section. Reasons for perinatal death are registered accurately in our database as this parameter is one of the most important for perinatal outcomes. Table 8 shows a slight increase in the total absolute number of perinatal deaths in 2008, but a non - significant relative decrease (6.2‰ versus 6.7‰ in 2000). In general, it can be said that the 3% rise in CS in the studied period did not result in a substantial increase of perinatal survival, although there were 8 more perinatal deaths due to asphyxia in 2000 when compared with 2008 (7.7% in 2000 versus 5.6% in 2008). The most remarkable changes in perinatal mortality are found in the categories 'intra - uterine death of a normal fetus' 111 infants in 2008 versus 88 in 2000) and 'low birth weight' (52 infants in 2008 versus 39 in 2000).

**Table 8:** Reasons for perinatal death in the SPE - population (2000, 2004 and 2008)

	2000	2004	2008	Δ	OR (95% CI)
**Total**	**418 (6.7‰)**	**398** **(6.4‰**)	**431 (6.2‰)**	**+ 13** **(- 0.5‰)**	**0.92 (0.80-1.06)**

Intra - uterine death of a normal fetus	21.1%	21.9	25.7%	+ 4.6	1.30 (0.93-1.81)

Congenital malformations	23.7%	25.8	24.8%	+ 1.1	1.06 (0.77-1.47)

Low birth weight	9.3%	11.7	12.0%	+ 2.7	1.33 (0.84-2.12)

Hypertension or other illness of the mother	1.0%	1.8	1.6%	+ 0.6	1.71 (0.45-6.99)

Abruptio placentae	7.9%	3.3	5.3%	- 2.6	0.66 (0.37-1.18)

Asphyxia/trauma of the child	7.7%	5.4	5.6%	- 2.1	0.71 (0.40-1.27)

Specified cause	15.0%	16.1	13.2%	- 1.8	0.86 (0.57-1.29)

Unknown	12.0%	14.0	11.3%	- 0.7	0.94 (0.61-1.47)

## Discussion

Our results illustrate that the rate of CS is particularly rising in the lowest risk population: term primiparae with a singleton in cephalic presentation. In 2008, the group of term nulliparous women with a singleton cephalic in spontaneous labor contributed as much to the CS - rate as the group with nulliparous breech presentations. The contribution of repeat CS to the overall rate was already considerable in 2000; however, in 2008 the contribution of this group is manifest. Since the incidence of abdominal delivery keeps rising in the low-risk group, this trend is not expected to turn. Furthermore, the proportion of inductions of labor decreased in favor of elective CS, in the meanwhile inductions of labor more often end in non-elective CS as well.

### The impact of changing trends in the patient population

Changing trends in mode of delivery may be a reflection of associated changes in the patient population. Assessment of our patient populations demonstrates increasing proportions of women of advanced age and elevated use of assisted reproduction. However, multiple regression analysis did not indicate these parameters to have a large impact on our results.

Our dataset did not allow to adjust for CS due to maternal request or the impact of maternal obesity, which is a shortcoming.

### The impact of hospital policy

Our comparison between the Flemish hospital with the lowest and highest abdominal deliveries shows that hospital policy has an essential impact on CS rate. A lot of studies consider a Caesarean section rate of 15% as the most optimal. This cut-off is based on the prevalence of maternal complications which could be effectively addressed by advanced levels of care including (but not limited to) Caesarean section [3]. This rate is recommended to ensure maternal safety, so maybe there should be space for discussion and reconsideration whether the 15% rate is not too narrow to guarantee both maternal and fetal safety. According to Robson (2001), the question is not whether CS rates are too high or too low, but rather whether abdominal deliveries are performed when appropriate or not in a given circumstance. For Robson, a CS is convenient when the information is available to explain and justify it [7]. Brennan et al. [9] compared Caesarean delivery rates between nine institution of different countries. Their results showed significant variability in overall CS rates across the centers. These variations were largely explained by variations in spontaneously laboring term nulliparae.

It would be of interest to account for obstetrician characteristics (age, experience in vaginal breech deliveries, etc.) in future studies.

### Complications of CS, in the aftermath and in future reproduction

Complications for the following pregnancy include a higher incidence of placenta previa and accrete (of which the risk is greater than 60% in women who have had four or more abdominal deliveries). On the shorter term, there are more thromboembolic problems and infections after CS [10]. Mother - child acquaintance is postponed after CS, in that standard admission to neonatal care is more common [3], which introduces to health - economical considerations. The health economical aspect of the abdominal delivery has escaped extended research so far, notwithstanding CS to be the operation most often performed in Western countries [11]. With longer hospital stays for both mother and child plus the additional complications, it may be assumed that abdominal delivery is the less economical option.

## Conclusion

Results of this study show that if we want to bring the rising CS rate to a halt, we must concentrate on low-risk primipare women. Refraining first elective sections will have an impact on repeat sections in the future as well. According to Scott (2002), elective CS may not be compared with other elective surgery, in that the latter does not influence the reproductive future of a patient. Minkoff et al. (2003) referred to the longer life expectancy of women and decreased child wish in order to promote (elective) CS. As women live longer, long - term complications such as pelvic floor problems are more important; while there is less concern about the risk of repeated procedures with a lower fertility rate. Such rationale may be a hazardous basis for professional guidelines, since fertility rates are not constant over time and borders. In Flanders, couples currently tend to have two children or more

Robson achieved success with his hospital - based audits in order to draw back the CS rate. In these audits, appropriateness of individual elective CS is discussed within the medical team. This mode of operation has been outlined by other authors as well [10,12]. The audit of the van Dillen study resulted in a 18.7% section rate (in total of 1221 deliveries), which was a 5% decrease compared with the period prior to the audit. In 24.4% of cases, discussion about necessity was carried out. Also the audits illustrated in the other studies, resulted in a lower CS rate.

Next to peer discussion about necessity, Robson recommends accurate registration of both numbers and indications for abdominal delivery whereby one main indication should be registered rather than a list of indications [7].

In conclusion, although elective Caesarean section may appear a safe option in the short term; the long term consequences should also be considered. Every elective abdominal delivery in a nullipare woman should be considered as a hallmark for her reproductive future and as such should be considered thoroughly and preferably in a discussion with peers.

## Competing interests

The authors declare that they have no competing interests.

## Authors' contributions

ID contributed in the interpretation of data and manuscript drafting. HC contributed to the study design, interpretation of data and manuscript revision. EM contributed in the study design, data acquisition, data analysis and manuscript revision. IT contributed in the study design, interpretation of data and manuscript revision. GM contributed in the study design, data acquisition, data analysis and manuscript revision. MT contributed to interpretation of data and manuscript revision. All authors agree for publication of this final version.

## Pre-publication history

The pre-publication history for this paper can be accessed here:

http://www.biomedcentral.com/1471-2393/12/3/prepub
